# The Seeding and Cultivation of a Tropical Species of Filamentous *Ulva* for Algal Biomass Production

**DOI:** 10.1371/journal.pone.0098700

**Published:** 2014-06-04

**Authors:** Christina Carl, Rocky de Nys, Nicholas A. Paul

**Affiliations:** MACRO – the Centre for Macroalgal Resources and Biotechnology, School of Marine and Tropical Biology, James Cook University, Townsville, Queensland, Australia; Auckland University of Technology, New Zealand

## Abstract

Filamentous species of *Ulva* are ideal for cultivation because they are robust with high growth rates and maintained across a broad range of environments. Temperate species of filamentous *Ulva* are commercially cultivated on nets which can be artificially ‘seeded’ under controlled conditions allowing for a high level of control over seeding density and consequently biomass production. This study quantified for the first time the seeding and culture cycle of a tropical species of filamentous *Ulva* (*Ulva* sp. 3) and identified seeding density and nursery period as key factors affecting growth and biomass yield. A seeding density of 621,000 swarmers m^-1^ rope in combination with a nursery period of five days resulted in the highest growth rate and correspondingly the highest biomass yield. A nursery period of five days was optimal with up to six times the biomass yield compared to ropes under either shorter or longer nursery periods. These combined parameters of seeding density and nursery period resulted in a specific growth rate of more than 65% day^−1^ between 7 and 10 days of outdoor cultivation post-nursery. This was followed by a decrease in growth through to 25 days. This study also demonstrated that the timing of harvest is critical as the maximum biomass yield of 23.0±8.8 g dry weight m^−1^ (228.7±115.4 g fresh weight m^−1^) was achieved after 13 days of outdoor cultivation whereas biomass degraded to 15.5±7.3 g dry weight m^−1^ (120.2±71.8 g fresh weight m^−1^) over a longer outdoor cultivation period of 25 days. Artificially seeded ropes of *Ulva* with high biomass yields over short culture cycles may therefore be an alternative to unattached cultivation in integrated pond-based aquaculture systems.

## Introduction

The global production of seaweeds (marine macroalgae) of 19 million tonnes is dominated by only five key species and their products are targeted for food and phycocolloid markets [1]. While less than 0.1% of total production is accounted for by green seaweeds [2], species within the genus *Ulva* have in many ways the most compelling characteristics for biomass applications in their cosmopolitan distribution [3], very high growth rates [4], high stocking densities [5], [6], and wide environmental tolerances for year round production [7], [8]. However, the existing commercial production of *Ulva* is either focussed on niche, high-value food products in Japan using controlled seeding with small production volumes (‘aonori’; ∼1,500 tonnes dry weight per annum; [9]) or on integrated aquaculture in land-based high rate algal ponds in South Africa using vegetative cultivation of biomass as abalone feed (∼1,000 tonnes fresh weight per annum; [10]). There are also many studies that discuss the suitability of *Ulva* as biomass energy resource including biogas [11], ethanol and other alcohols [12] and biocrude [13]. In addition, the application of *Ulva* as a feedstock for nutraceuticals, in particular sulphated polysaccharides (ulvans), has been investigated [14], [15]. To ensure a reliable and cost-effective biomass supply of *Ulva*, it is essential to develop and optimise the closed-life cycle production and yield of biomass, as a fundamental basis for commercial applications and more widespread production throughout the globe.

The genus *Ulva* can be categorised as having either a blade-like or filamentous (syn. *Enteromorpha*; [16]) morphology. In general, both morphologies can tolerate a wide range of environmental conditions and have high growth rates [8], that can result in the formation of algal blooms in eutrophic coastal waters (‘green tides’). However, filamentous species, in particular, can have exceptional environmental tolerance and can grow in salinities ranging from 10 to 60 ppt [7] and temperatures up to 40°C [17]. Therefore, filamentous species of *Ulva* are ideal for cultivation across a broad range of environments and can be integrated into existing aquaculture production thereby providing value-adding processes and environmental services by removing dissolved nutrients from waste waters [18].

Filamentous species of *Ulva* can be maintained in free-floating cultures, either as ‘germling clusters’ [19] or using surface barriers [18], [20]. However, many highly productive species of filamentous *Ulva* require a closed-life cycle with the production of seedlings and efficient seeding and cultivation techniques for the intensive production of biomass. For example, species of filamentous *Ulva* used in in the Asian cuisine, ‘aonori’ [21], have been cultivated since 1985 in Japan using nets. Traditionally, seedlings have been obtained from natural settlement by submerging nets in calm areas with naturally high abundances of the desired species [9]. Spores released from natural populations then settle on the nets which are subsequently transferred to cultivation grounds [22]. However, natural seeding provides for little control over the density of seeding and distribution. In contrast, artificial seeding allows for a high level of control and seeding techniques have been investigated since the 1980s [23], [24].

The practise of artificial seeding relies heavily on manipulative treatments to induce reproduction independent of seasonal reproduction patterns. A reliable method for artificially inducing reproduction with high reproductive output has been successfully demonstrated for the filamentous species of *Ulva*, *Ulva* sp. 3 [25]. This species has only recently been described [26] and its distribution ranges from the East coast of Australia [8] to Southern Japan [26]. *Ulva* sp. 3 is a robust species with high growth rates across a wide range of temperatures [8] and a salinity tolerance from brackish [26] to saline [8], making it ideal for cultivation for biomass applications. In order to achieve commercially viable cultivation of this target species, fundamental data on the optimal density of seeding and nursery period are first required, followed by an understanding of how these initial conditions influence growth at scale. Seeding density is directly related to intraspecific competition for resources, particularly light and nutrients, which can result in reduced growth and increased mortality [27]–[29]. Similarly, the nursery period is critical to minimise the detachment and loss of germlings due to hydrodynamic forces [30]. Notably, an increased contact time with the surface enhances overall settlement [31] and the adhesion strength of settled spores [32].

The aim of this study was therefore to establish optimum density of settled swarmers and nursery conditions for *Ulva* sp. 3 that results in high biomass yields. To determine the optimal seeding density for *Ulva* sp. 3, ropes were artificially seeded under controlled conditions using swarmers (collective term for motile reproductive bodies, including gametes, zoospores, zoids). The seeding densities ranged from 155 to 1,552×10^3^ swarmers m^−1^ rope. In addition, nursery periods from one to ten days were tested for theses densities and subsequently, the seeded ropes were maintained in outdoor tanks for 21 days to determine weekly growth rates. Finally, ropes were seeded at the optimal density and were, after the optimum nursery period of five days, maintained in outdoor tanks for up to 25 days to determine biomass yields, growth rates, fresh weight to dry weight ratio (FW:DW), ash content, and the morphology of filaments over this period.

## Materials and Methods

### Collection of algal biomass and preparation of reproductive material

The species used in this study was *Ulva* sp. 3 [26] and identified using molecular barcoding [8], [25]. *Ulva* sp. 3 (hereafter *Ulva*) was characterised by flat tubular thalli and collected in the morning by hand from a land-based aquaculture facility at Guthalungra (19°55′S, 147°50′E), Queensland, Australia. Permission was obtained from owners to collect algae from this site. Samples were placed in a 25 L container filled with pond water and then transported within 3 h to the laboratory at James Cook University in Townsville, Australia. Subsequently, the samples were gently washed three times with filtered seawater (FSW; 0.2 µm and UV sterilised) to remove debris, epiphytes and invertebrates. To induce the release of swarmers, *Ulva* was shocked at a temperature of 4°C for 10 min [25] and subsequently chopped using a blender in the early afternoon on the day of collection. The chopped filaments were retained with a sieve (120 µm), washed with autoclaved FSW and subsequently placed in a crystallising dish filled with approximately 200 mL autoclaved FSW. Approximately 0.5–1 mL of this suspension was then transferred in Petri dishes filled with 10 mL autoclaved FSW using a transfer pipette and subsequently placed in a temperature controlled culture cabinet (Sanyo MLR-351) at 25°C at an irradiance of 125 µmol photon m^−2^ s^−1^ under a 12 h L: 12 h D photoperiod to maximise the release of swarmers [25]. The release of swarmers peaked after two days between 10:00 and 11:30 am. The Petri dishes were then emptied in a beaker through a sieve (120 µm) to filter out the chopped filaments, while the released swarmers were collected with the seawater in the beaker. The density of the swarmers was calculated using a haemocytometer.

### Optimal seeding density and nursery period

To determine which seeding density and nursery period resulted in the highest biomass yield and growth, swarmers were seeded onto polypropylene ropes (Ø 4 mm, Syneco) at densities of 155, 310, 466, 621, and 1,552×10^3^ swarmers m^−1^ rope. A total of 45 pieces of rope were seeded, each with a length of 580 mm. These were attached to the bottom of individual Petri dishes (Ø 60 mm, Techno Plas; S6014S10) as a flat spiral coil using the pressure-sensitive adhesive Ezy Tack (Selleys). The seeding density was altered by manipulating the density of swarmers to 5, 10, 15, 20, and 50×10^3^ swarmers mL^−1^ and then adding 18 mL of this swarmer suspension to each Petri dish with a piece of rope using a syringe. The tested nursery periods were 1, 5, and 10 days and the Petri dishes were kept in a culture cabinet (Sanyo MLR-351) in a 12 h L:12 h D photoperiod at 25°C during this period. The water was changed, where applicable, after 3, 5, and 8 days. There were three replicates (*n* = 3) for each seeding density for each tested nursery period. Subsequent to the time in the nursery, the seeded ropes were carefully removed from the Petri dishes using tweezers, uncoiled and transferred to aerated flow-through outdoor tanks under ambient light at the Marine & Aquaculture Research Facilities Unit (MARFU).

A total of 5 seeded ropes (one from each density) were attached to a weighted frame (380×500 mm) using cable ties. Each frame was placed on the bottom of a tank (*n* = 9 replicate tanks) so that the ropes were immersed horizontally in the water at a depth of approximately 100 mm below the surface. To minimise temperature fluctuations, the tanks holding the ropes were placed in a circulating water bath (see cultivation conditions following). After 7, 14, and 21 days of outdoor cultivation, the tanks, frames and air lines were cleaned and the seeded ropes were repeatedly sampled to measure the fresh weight (FW) of *Ulva*. The ropes were spun dry and weighed (FW) using a digital balance (Kern KB 3600-2N). After 21 days of outdoor cultivation, the seeded ropes were dried in an oven at 65°C for at least 7 days to determine the dry weight (DW) of *Ulva*. The fresh and dry biomass yield was calculated as FW (g FW m^−1^ rope) and DW (g DW m^−1^ rope) per linear meter of rope, respectively. Individual FW:DW ratios were calculated for each replicate. Specific growth rate was calculated for each replicate using the equation SGR (% day^−1^) * =  ln (B_2_/B_1_)/ (t_2_–t_1_) · 100*, where *B_1_* and *B_2_* are the biomasses (g FW) at time *t_1_* and *t_2_* of outdoor cultivation (days).

Experiments were conducted during the Australian winter with an average water temperature of 25°C and the seeded ropes received on average photosynthetically active radiation of 29.2 (±6.4 S.D.) mol photons m^−2^ day^−1^. The tanks were on a recirculating system with an average nitrate-N and phosphorous concentration of 2.09 (±1.47 S.D.) mg NO_3_-N L^−1^ and 0.37 (±0.22 S.D.) mg P L^−1^, respectively. Each tank held approximately 28 L of FSW (1 µm) and had a flow rate of 0.5 L min^−1^. The average salinity was 31.4 (±1.4 S.D.) ppt.

### Biomass yield and growth of *Ulva* seeded at optimal seeding density and nursery period

To determine the dry biomass yield, growth, FW:DW ratio, ash content and change in morphology over time, ropes were seeded at an optimal density of 621,000 swarmers m^−1^ rope and maintained for five days under nursery conditions (see results *‘Optimal seeding density and nursery period’*). The arrangement of ropes in Petri dishes, and the seeding and nursery procedure, followed the methods described in the previous experiment. After five days under nursery conditions, the seeded ropes were removed from the Petri dishes and transferred to aerated flow-through outdoor tanks at MARFU as described above. Each seeded rope was individually attached to a weighted frame placed on the bottom of a tank holding approximately 28 L of FSW (1 µm and UV sterilised) for 25 days. Holding tanks, frames and air lines were cleaned after 7, 13, and 19 days. The seeded ropes were destructively sampled (*n* = 3) after 7, 10, 13, 16, 19, 22, and 25 days of outdoor cultivation and photographed with a digital camera (Canon PowerShot D20) to determine the width of filaments (*n* = 50 per samples) using Image J freeware (www.nih.giv). Subsequently, samples were spun dry and weighed to determine the growth (SGR) using the equation above where *B_1_* and *B_2_* are the average biomasses (g FW). Thereafter, the samples were dried in an oven at 65°C for at least 7 days to quantify the DW and to calculate the dry biomass yield as DW per linear meter of rope (g DW m^−1^ rope). FW:DW ratios were calculated as described above. The ash content of each replicate was quantified by heating a 1.5 g homogenised subsample of dried biomass at 110°C in a moisture balance until a constant dry weight was reached and then combusting at 550°C in a muffle furnace for 24 h until a constant weight was reached.

Experiments were conducted with a total of three independent sample collections of *Ulva* as initial parent generation used as a source of swarmers to seed the ropes during the Australian spring (hereafter referred to as ‘batch’). The water temperatures in the tanks ranged between a night time minimum of 21.7°C and a daytime maximum of 36.4°C during the trial, with an average water temperature of 27.8 (±3.0 S.D.)°C. The average salinity was 32.7 (±0.9 S.D.) ppt and the seeded ropes received an average photosynthetically active radiation of 47.0 (±6.8 S.D.) mol photons m^−2^ day^−1^ during the experiment. The tanks had a flow rate of 0.5 L min^−1^ and were on a recirculating system with an average concentration of nitrogen as nitrate of 1.50 (±1.89 S.D.) mg NO_3_-N L^−1^ and an average concentration of phosphorous of 0.40 (±0.25 S.D.) mg P L^−1^.

### Statistical analysis

Data were analysed by permutational analysis of variance (PERMANOVA) using PRIMER 6 (v. 6.1.13) and PERMANOVA+ (v. 1.0.3) [33]. The Bray-Curtis dissimilarity measure was used for all PERMANOVAs and *p*-values were calculated using permutation of residuals under a reduced model with 9999 random permutations. If there was a significant difference, pair-wise *a posteriori* comparisons were made among the significant groups using the Bray-Curtis similarity measure (α = 0.05). All data are reported as mean ±1 standard error (S.E.) unless stated otherwise.

To formally test the effect of seeding density on the biomass yields (fresh and dry) after 21 days of outdoor cultivation for each nursery period in the first experiment, density was considered as a fixed factor and tank as an unreplicated blocking factor as seeded ropes in one tank were not independent. To determine the effect of nursery period and sampling day on the fresh biomass yields of seeded ropes, the optimal seeding density of 621,000 swarmers m^−1^ (see results) was selected for PERMANOVA analysis and nursery was considered as fixed and sampling day as random factors. To analyse the effect nursery period on the dry biomass yield after 21 days of outdoor cultivation of ropes seeded at 621,000 swarmers m^−1^, data was analysed using an one-factor PERMANOVA. To test for the effect of sample collection on the dry biomass yield, FW:DW ratio, SGR, ash content, and width of filaments over time in the second experiment, two-factor PERMANOVAs were used with time as a fixed factor (with destructively sampled replicates) and sample collection as a random factor. FW:DW ratios for both experiments were correlated with dry biomass yield for each replicate using the software Statistica (v.12).

## Results

### Optimal seeding density and nursery period

**Figure 1:**
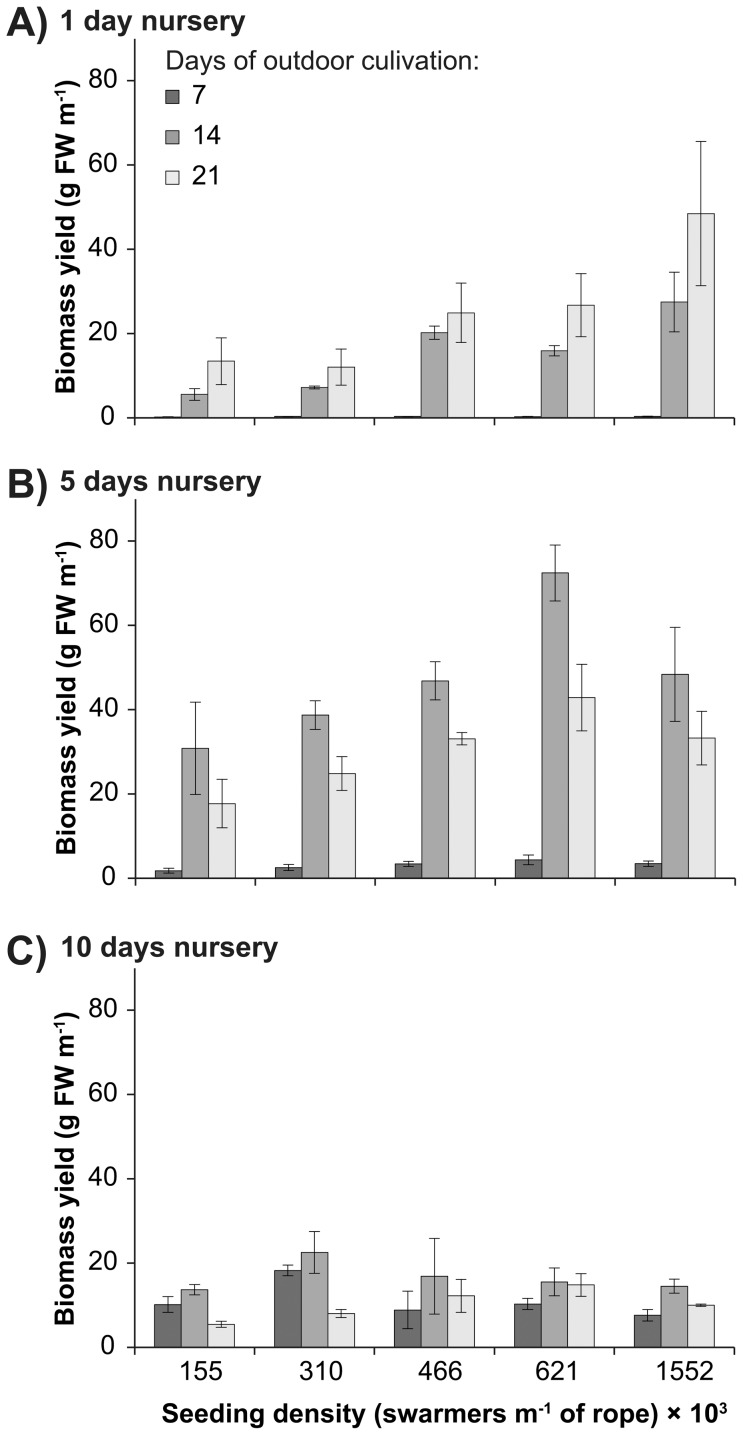
Fresh biomass yield. Mean (± S.E.) biomass yield (g FW m^−1^ rope) of *Ulva* seeded onto ropes at densities from 155 to 1,552×10^3^ swarmers m^−1^ rope. Seeded ropes were maintained in a nursery for a period of (**a**) one, (**b**) five, and (**c**) ten days prior to transfer to outdoor cultivation. The fresh biomass yield was quantified after 7, 14, and 21 days of outdoor cultivation.

The highest fresh biomass yields were obtained for ropes maintained for five days in the nursery, regardless of seeding density, and had approximately two to six times the biomass than ropes maintained under shorter and longer nursery periods (Figure 1). The highest fresh biomass yield was obtained for the seeding density of 621,000 swarmers m^−1^ rope after the nursery period of five days, and this density was nearly double the yield obtained at any other seeding density and nursery period (Figure 1). Therefore, this seeding density in combination with a five day nursery period was chosen for the subsequent experiment on the basis of biomass yield. When specifically analysing the fresh biomass yield of ropes seeded at 621,000 swarmers m^−1^ rope to identify differences between nursery periods over time within a single seeding density, there was a significant interactive effect between nursery and days of outdoor cultivation, driven by significant differences between nursery periods and lower biomass yields overall after day 7 in comparison to day 14 and 21 (Table 1).

**Table 1:** PERMANOVA analysis on Bray-Curtis distances testing the effects of nursery period (Nursery, fixed factor) and days of outdoor cultivation (Day, random factor) on the fresh biomass yield of ropes seeded at 621×10^3^ swarmers m^−1^ rope; and the effect of nursery on the dry biomass yield after 21 days outdoor cultivation.

		Biomass yield per metre rope					
		Fresh biomass			Dry biomass		
*Source*	*df*	*MS*	*F*	*P*	*MS*	*F*	*P*
Nursery	2	5552	1.26	0.363	1015	2.61	0.106
Day	2	7760	26.58	**<0.001**	*No test*		
Nursery × Day	4	4423	15.15	**<0.001**	*No test*		

Mean square (*MS*), pseudo-F (*F*) and *P* values are presented, significant terms shown in bold.

After the shortest nursery period (one day), the average fresh biomass yields increased for all seeded ropes over 21 days and also increased with increasing seeding densities (Figure 1a). The lowest biomass yields were observed after seven days and were below 0.4 g FW m^−1^ rope for all seeding densities. In the following days, the yields increased more than 10-fold and ranged from 5.5±1.4 g FW m^−1^ rope to 27.5±7.1 g FW m^−1^ rope after 14 days and from 12.1±4.3 g FW m^−1^ rope to 48.5±17.1 g FW m^−1^ rope after 21 days (Figure 1a). The highest seeding density of 1,552×10^3^ swarmers m^−1^ rope had a greater biomass yield than lower seeding densities after a nursery period of one day, despite some variability between tanks in the analysis (Table 2).

**Table 2:** PERMANOVA analysis on Bray-Curtis distances testing the effects of seeding density (Density, fixed factor) on the (**a**) fresh (g FW m^−1^ rope) and (**b**) dry biomass yield (g DW m^−1^ rope) of seeded ropes at each nursery period in an unreplicated blocked design (Tank: blocked factor) after 21 days outdoor cultivation.

		Biomass yield per metre rope					
		1 day nursery		5 days nursery		10 days nursery	
*Source*	*df*	*F*	*P*	*F*	*P*	*F*	*P*
**(a) Fresh biomass**							
Density	4	2.35	0.053	2.64	0.086	3.00	0.075
Tank	2	5.91	**0.003**	0.67	0.576	1.24	0.341
**(b) Dry biomass**							
Density	4	2.77	**0.030**	2.16	0.130	4.01	**0.030**
Tank	2	4.85	**0.006**	0.41	0.757	0.60	0.620

Pseudo-F (*F*) and *P* values are presented, significant terms shown in bold.

After the nursery period of five days, the biomass yields were generally low after seven days and increased approximately 16-fold during the following days, ranging from 30.8±10.9 g FW m^−1^ rope to 72.5±6.6 g FW m^−1^ rope at day 14. Subsequently, the yields decreased after 21 days and ranged from 17.7±5.8 g FW m^−1^ rope to 42.9±7.9 g FW m^−1^ rope (Figure 1b).

After the longest nursery period (ten days), the biomass yields ranged from 10.1±1.9 g FW m^−1^ rope to 18.2±1.3 g FW m^−1^ rope after seven days (Figure 1c) and were generally higher than for shorter nursery periods. However, the biomass yields overall were low for the following days and below 23 g FW m^−1^ rope at all times, which is less than half the yield than that of shorter nursery periods.

**Figure 2:**
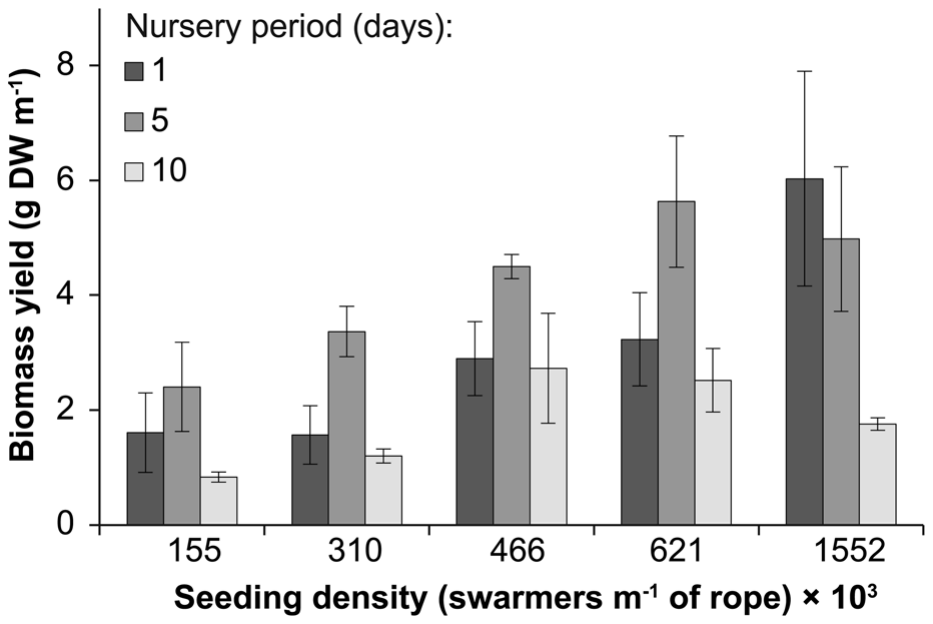
Dry biomass yield. Mean (± S.E.) biomass yield (g DW m^−1^ rope) of *Ulva* seeded onto ropes at densities ranging from 155 to 1,552×10^3^ swarmers m^−1^ rope after 21 days of outdoor cultivation. The nursery periods of the seeded ropes ranged from one to ten days.

Similar to the fresh biomass yields, the dry biomass yields overall were generally higher for ropes maintained at a nursery period of five days (4.2±0.5 g DW m^−1^ rope) compared to shorter (3.1±0.6 g DW m^−1^ rope) and longer nursery stages (1.8±0.3 g DW m^−1^ rope) (Figure 2). The average dry biomass yields ranged from 2.4±0.8 g DW m^−1^ rope to 5.6±1.2 g DW m^−1^ rope for a nursery period of five days, and were halved for a longer nursery period of ten days where yields ranged from 0.8±0.1 g DW m^−1^ rope to 2.7±1.0 g DW m^−1^ rope. Notably, the dry biomass yields were measured after 21 days of outdoor cultivation when the biomass on ropes maintained under a nursery period of five days had already degraded, on contrast to the shorter nursery period of one day which continued to increase. Nursery period had no significant effect when specifically analysing the dry biomass yield of ropes seeded at a single seeding density (Table 1). The dry biomass yield increased nearly 3-fold from the lowest seeding density of 155,000 swarmers m^−1^ rope (1.6±0.4 g DW m^−1^ rope) to the highest seeding density of 1,552×10^3^ swarmers m^−1^ rope (4.3±0.9 g DW m^−1^ rope) (Figure 2) with significant differences in dry biomass yield between seeding densities for nursery periods of one and ten days (Table 2). The average FW:DW ratios were highly variable ranging from 4.7±1.0 to 8.6±0.3 and generally decreased with increasing nursery periods with no consistent trend between FW:DW ratio and seeding density. There was no correlation between the FW:DW ratio and the dry biomass yield (*r* = 0.185, *p* = 0.224 ).

**Figure 3:**
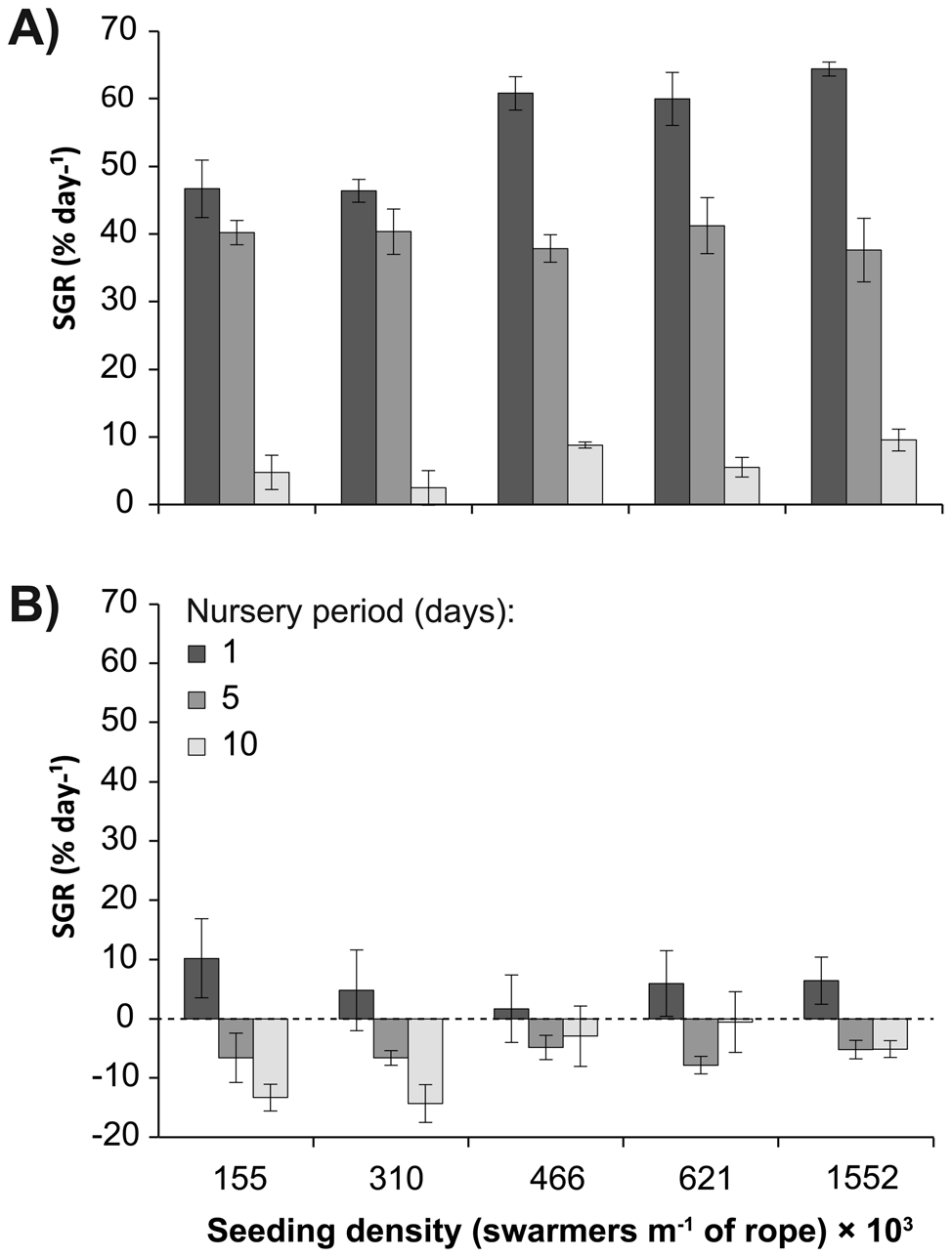
Specific growth rates. Mean (± S.E.) specific growth rate (SGR, % day^−1^) of *Ulva* seeded onto ropes at densities ranging from 155 to 1,552×10^3^ swarmers m^−1^ rope in the time period of (**a**) 7 to 14 days, and (**b**) 14 to 21 days of outdoor cultivation.

In contrast to fresh and dry biomass yields, specific growth rates were highest for the shortest nursery period of one day across all seeding densities because of the low initial biomass for each measurement (Figure 3). The average growth rates for *Ulva* maintained at a nursery period of one day ranged from 46.4±1.7 % day^−1^ to 64.4±1.0 % day^−1^ between 7 and 14 days of outdoor cultivation, while those maintained under the longest nursery period of ten days ranged from 2.4±2.6 % day^−1^ to 9.5±1.6 % day^−1^ (Figure 3a). The growth rates between 14 and 21 days of outdoor cultivation had substantially lower growth rates with the shortest nursery period being the only treatment with positive values (Figure 3b).

### Biomass yield and growth of *Ulva* seeded at optimal seeding density and nursery period

**Figure 4:**
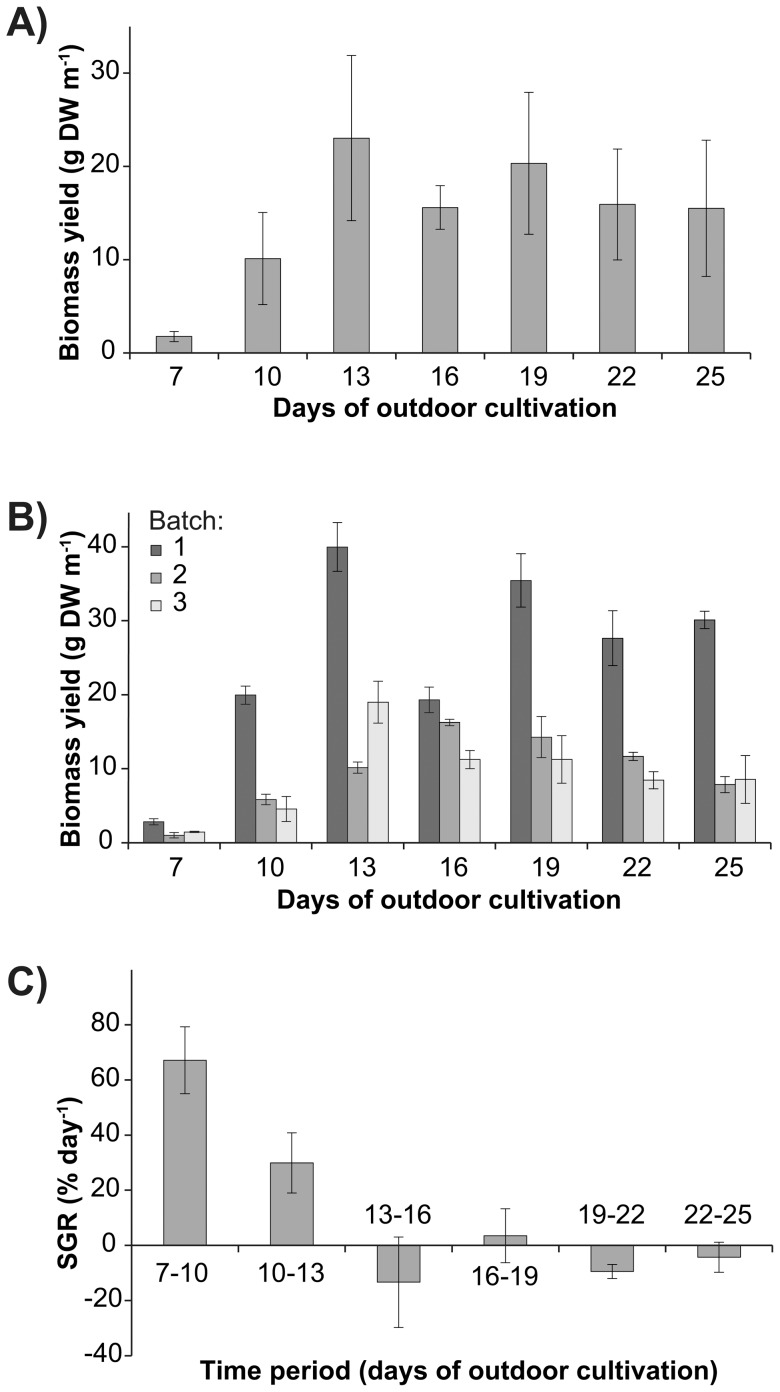
Mean dry biomass yields and growth rates of *Ulva* seeded onto ropes over time. Experiments were run at an optimal seeding density of 621×10^3^ swarmers m^−1^ rope and maintained for five days under nursery conditions. (**a**) Mean (± S.E.) dry biomass yield (g DW m^−1^ rope) over time from three independently collected batches of algal biomass (*n* = 3 for each batch). Algal batches were collected on 11 September 2013 (batch 1), 18 September 2013 (batch 2), and 1 October 2013 (batch 3). (**b**) Mean (± S.E.) dry biomass yield (g DW m^−1^ rope) over time (*n* = 3) for each batch. (**c**) Mean (± S.E.) specific growth rate (SGR, % day^−1^) over time (*n* = 3).

The average dry biomass yield increased more than 10-fold from day 7 (1.8±0.5 g DW m^−1^ rope) to day 10 (10.1±4.9 g DW m^−1^ rope) and then day 13 (23.0±8.8 g DW m^−1^ rope) (Figure 4a). Subsequently, the biomass yield decreased to below 20.4 g DW m^−1^ rope for extended time periods of outdoor cultivation. There was a significant interaction of batch and days of outdoor cultivation (Table 3), driven by differences in the time when the highest biomass yield was attained among batches (Figure 4b). While the biomass yield peaked at day 13 for the first batch (40.0±3.3 g DW m^−1^ rope) and third batch (19.0±2.8 g DW m^−1^ rope), the second batch had the highest biomass yield at day 16 (16.2±0.4 g DW m^−1^ rope). Furthermore, there were striking differences in the biomass yield between batches; however, batches showed a similar trend of an initial increase and subsequent decrease in dry biomass yield over time (Figure 4b). Notably, the biomass matured and was reproductive during the course of the experiment and the release of swarmers occurred after 13 days of outdoor cultivation. Average specific growth rates decreased significantly over time (Figure 4c, Table 3) with the highest growth rate between 7 and 10 days of outdoor cultivation (67.1±12.2 % day^−1^). There was a subsequent reduction in growth rate between 10 and 13 days (29.9±10.9 % day^−1^). After 13 days of outdoor cultivation, growth rates were generally lower and ranged from −13.4±16.4 % day^−1^ (days 13 to 16) to 3.5±9.8 % day^−1^ (days 16 to 19).

**Table 3:** PERMANOVA analysis on Bray-Curtis distances testing the effects of days of outdoor cultivation (Day, fixed factor) and batch (Batch; random factor) on the biomass yield per metre rope (Fresh biomass; Dry biomass), FW:DW ratios (FW:DW), width of filaments (Width), ash content (Ash) and specific growth rate (SGR) of *Ulva*.

		Dry biomass yield		FW: DW		Width		Ash	
*Source*	*df*	*F*	*P*	*F*	*P*	*F*	*P*	*F*	*P*
Day	6	5.5	0.001	2.6	0.65	16.69	<0.001	3.3	0.025
Batch	2	34.2	<0.001	51.1	<0.001	51.2	<0.001	39.42	<0.001
Day × Batch	12	5	<0.001	6.1	<0.001	18.75	<0.001	2.5	0.006
		**SGR**							
Source	df	F	P						
Day	5	7.47	0.005						
Batch	2	0.19	0.828						
Day × Batch	10	*No test*							

Pseudo-F (*F*) and *P* values are presented.

**Figure 5:**
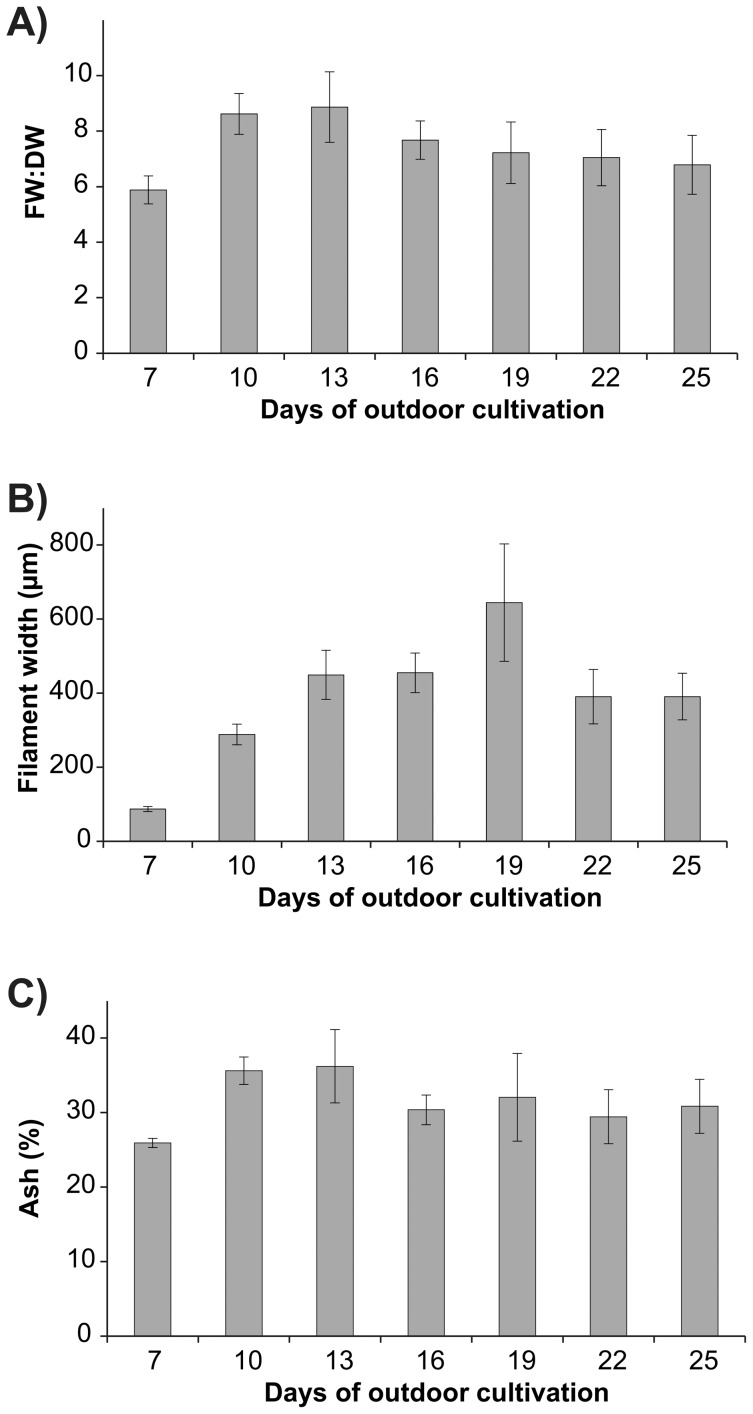
Mean FW:DW ratios, filament width and ash content of *Ulva* seeded onto ropes over time. Experiments were run at an optimal seeding density of 621×10^3^ swarmers m^−1^ rope and maintained for five days under nursery conditions. (**a**) Mean (± S.E.) FW:DW ratio (*n* = 3). (**b**) Mean (± S.E.) width of filaments of *Ulva* growing on ropes over time (*n* = 3). (**c**) Mean (± S.E.) ash content (%) of *Ulva*.

Average FW:DW ratios ranged from 5.9±0.5 (day 7) to 8.9±1.3 (day 13) (Figure 5a) and overall patterns were generally variable between batches, reflected by a significant interaction between batch and days of outdoor cultivation (Table 3). In addition, FW:DW ratios were positively correlated with an increase in dry biomass yield (*r* = 0.788, *p*<0.001). The average width of filaments increased more than 7-fold from day 7 (86.7±7.0 µm) to day 19 (644.3±158.4 µm) (Figure 5b). Subsequently, the average width decreased to 390.3±73.7 µm and 390.6±63.0 µm at days 22 and 25, respectively. The ash content ranged from 25.9±0.6 % (day 7) to 36.2±4.9 % (day 13) (Figure 5c).

## Discussion

This study demonstrates the importance of seeding density and nursery period on the biomass yield and growth rate of *Ulva* sp. 3 when artificially seeded onto ropes. Optimal cultivation conditions resulting in the highest biomass yields were achieved at a seeding density of 621,000 swarmers m^−1^ rope and a nursery period of five days. Seeding density was a key factor affecting both growth rate and biomass yield of *Ulva* sp. 3 confirming previous studies, where the biomass and size of seaweeds was related to their settlement density [27], [30], [34]. In general, the density of germlings is negatively related to growth and survival due to intraspecific competition and shading effects [27], yet low seeding densities on ropes can result in higher growth of epiphytes and thus high seeding densities are also a strategy to control biofouling on cultured seaweeds [35]. However, this might be less critical for fast-growing and opportunistic species such as filamentous *Ulva*, meaning that the selection of seeding density can be based on controlled cultivation conditions. In addition, the optimal seeding density for *Ulva* of 621,000 individuals m^−1^ rope, as quantified in this study, is generally much higher than for other species, where common seeding densities are 2,000 individuals m^−1^ rope for the brown seaweeds *Undaria pinnatifida* [36] and *Sargassum fulvellum* [37], 2,000 to 3,000 individuals m^−1^ rope for *Saccharina latissima* [38], [39], and 20,000 individuals m^−1^ rope for the red seaweed *Gracilaria chilensis* [40]. Furthermore, brown and red seaweeds typically have longer nursery and culture cycles with correspondingly higher productivities expected per linear metre (∼7 kg FW m^−1^ rope after 5 months for *G. chilensis*; [40]). This highlights the differences in the morphology and cultivation techniques of commercial brown and red seaweeds compared to filamentous *Ulva*, with clear distinction in the length of production cycle.

A further key factor affecting growth and biomass yield of *Ulva* was the nursery period prior to grow-out. This period is critical for the success of viable mass-cultivation of *Ulva* as an increased contact time acts to minimise detachment and loss of germlings due to hydrodynamic forces [30]. This effect is consistent with the difference in fresh biomass yields for seeded ropes maintained under nursery conditions for one and five days where values were lower for the shorter nursery period, most probably due to higher detachment of settled germlings after only one day. Interestingly, an extended nursery period of ten days had adverse effects on the growth of *Ulva* with the lowest biomass yields and growth rates overall among nursery periods. This implies that resources may be limited under static nursery conditions [41], [42] resulting in poor growth performance of the germlings.

The trend of initially increasing and then decreasing biomass yield, coupled with decreasing growth rates over time, is likely due to a general decrease of light availability with increasing biomass on the ropes [43]. Furthermore, the decline in growth of *Ulva* sp. 3 also reflects the maturation of biomass and the reproductive events after 13 days of outdoor cultivation. This short life cycle is characteristic of opportunistic species and is notably also shorter than other species of *Ulva*, including *U*. *ohnoi* (∼3 weeks; [44]) and tropical *U. flexuosa* (∼4 weeks; [34]), and much shorter than the life cycle for other seaweed species (*Sargassum horneri*; ∼4.5 months; [45]). Vegetative cells of *Ulva* transform directly into reproductive cells and, therefore, maturation and reproduction results in the degradation of filaments once the swarmers have been released. As such, the timing of harvest is critical to maximise yields. Furthermore, it is also important if mature populations on the ropes are to be used as parental stock for future generations through artificial seeding.

The species *Ulva* sp. 3 is an ideal candidate for biomass applications with its high biomass yields and growth rates as shown in this study, and its ability to integrate with existing aquaculture facilities [8], which together promise to make algal cultivation for biomass applications more cost-effective. The specific growth rate was high at more than 65% day^−1^ which is higher than recorded in a previous laboratory study (∼30% day^−1^; [8]). Overall, the growth rate was higher than for other species of *Ulva* and macroalgae, such as *U. reticulata* (∼4% day^−1^; [46]), *U. ohnoi* (∼38% day^−1^;[4]), *Cladophora* and *Chaetomorpha* (∼44% day^−1^; [47]), *Saccharina latissima* (∼5% day^−1^; [48]), and *Porphyra linearis* (∼16% day^−1^; [49]). Furthermore, *Ulva* sp. 3 was a robust species in this study tolerating varying environmental conditions across the experimental period. Water temperatures reached up to 36.4°C, without any signs of degradation of biomass, and other species of filamentous *Ulva* can be grown in water temperatures up to 40°C [17]. In addition, filamentous species of *Ulva* generally have a broad tolerance towards a wide range of salinities, including as low as 5 psu over 7 days, without a significant decrease in the viability of cells [50]. This supports *Ulva* sp. 3 as a promising candidate for year-around cultivation in tropical environments which are typically characterised by strongly seasonal rainfall providing a challenge for the consistent mass-cultivation of less robust algal species [51]. Finally, *Ulva* sp. 3 is a native species common at land-based aquaculture facilities in Eastern Australia and could therefore also be considered for bioremediation of waste waters from land-based aquaculture [8]. We suspect that similar results will be achievable for other filamentous species of *Ulva* with similar seeding density and nursery period based on similarities in morphology and size between species.

In conclusion, this study highlights the importance of controlling seeding densities in culture with large effects of doubling of the biomass yield at an optimal range per length of rope or potentially any unit of culture infrastructure. Therefore, ropes artificially seeded at an optimal density under controlled conditions will importantly maximise yields with an efficient use of the resource inputs and this provides a fundamental step in the cultivation of filamentous *Ulva* at scale. A reliable source of biomass for year-round production through optimised hatchery and culture cycles, in line with maximising biomass production per unit effort, allows for a reliable bioremediation strategy and further permits the development and management of selected breeding lines (genotypes). This study also revealed striking differences in the biomass yield between batches and underlines the need to select genotypes with high productivity for the cultivation of this species and to test the potential interactive effects with environmental conditions. Finally, populations seeded onto ropes can be used as parental plants for seedling production, which in turn reduces the need to harvest wild stocks.
